# Diffuse midline glioma of the cervical spinal cord with H3 K27M genotype phenotypically mimicking anaplastic ganglioglioma: a case report and review of the literature

**DOI:** 10.1007/s10014-020-00365-z

**Published:** 2020-05-25

**Authors:** Theo F. J. Kraus, Lukas Machegger, Johannes Pöppe, Barbara Zellinger, Eva Dovjak, Hans U. Schlicker, Christoph Schwartz, Barbara Ladisich, Mathias Spendel, Michael Kral, Annekathrin Reinhardt, Peter A. Winkler, Karl Sotlar

**Affiliations:** 1 Institute of Pathology, University Hospital Salzburg, Paracelsus Medical University, Müllner Hauptstraße 48, 5020 Salzburg, Austria; 2 Institute of Neuroradiology, University Hospital Salzburg, Paracelsus Medical University, Ignaz-Harrer-Str. 79, 5020 Salzburg, Austria; 3 Department of Neurosurgery, University Hospital Salzburg, Paracelsus Medical University, Ignaz-Harrer-Str. 79, 5020 Salzburg, Austria; 4 Department of Neuropathology, University Heidelberg, and CCU Neuropathology, DKFZ, Im Neuenheimer Feld 224, 69120 Heidelberg, Germany

**Keywords:** Anaplastic ganglioglioma, Diffuse midline glioma, *H3F3A* K27M, TERT C228T, *PDGFRα* Y849C, Methylome analysis

## Abstract

Here, we report on a 28-year old male patient presenting with neck and shoulder pain, dysesthesia of all four limbs and hypesthesia of both hands, without motor deficits. Magnetic resonance imaging showed an intradural, intramedullary mass of the cervical spinal cord of 6.4 cm length and 1.7 cm diameter. The patient underwent surgical resection. Histological and immunohistochemical evaluation showed pleomorphic glial tumor cells, mitoses, calcifications, and atypical ganglioid cells compatible with the morphology of anaplastic ganglioglioma (WHO Grade III). Extensive molecular workup revealed *H3F3A* K27M, TERT C228T and *PDGFRα* Y849C mutations indicating poor prognosis. The *H3F3A* K27M mutation assigned the tumor to the molecular group of diffuse midline glioma (WHO Grade IV). Epigenome-wide methylation profiling confirmed the methylation class of diffuse midline glioma. Thus, this is a very rare case of malignant glioma with H3 K27M genotype phenotypically mimicking anaplastic ganglioglioma. This case emphasizes the importance of comprehensive morphological and molecular workup including methylome profiling for advanced patient care.

## Introduction

In the 2016 revision of the World Health Organization (WHO) classification of tumors of the central nervous system (CNS) molecular genomic and epigenomic hallmarks were integrated into the classification and diagnosis of several brain tumors [1]. This development was the consequence of previous studies that revealed distinct methylation profiles defining novel entities [2–7]. One of the most prominent examples is the entity “diffuse midline glioma H3 K27M mutant” [1], typically arising within midline structures and the brain stem in younger patients [2]. Since these tumors show a devastating prognosis, they are assigned to WHO grade IV [1]. The characteristic hallmark of diffuse midline gliomas is the unique genotype of the tumors carrying the K27M mutation within one of the histone genes *H3F3A*, *HIST1H3B* or *HIST1H3C*.[2, 8, 9] In contrast to the nearly invariant mutations, these tumors show a highly variable histological phenotype mimicking numerous other tumors of the CNS such as glioblastoma, primitive neuroectodermal tumors, medulloblastomas but also low-grade gliomas [2, 9].

Gangliogliomas are rare glioneuronal tumors accounting for approximately 1.3% of all primary brain tumors [1]. They are frequently associated with epilepsy and are commonly located within the temporal lobe in younger patients [1]. Molecular genetic testing revealed that the *BRAF* V600E mutation is frequently found in gangliogliomas [1]. Since these tumors show a relatively benign behavior and malignant progression is rare, they are assigned to WHO grade I [1]. However, in 3–5% of all gangliogliomas anaplastic variants can be found: [1, 10–15]. In these cases, the glial component shows signs of anaplasia, increased proliferation activity and an increased mitotic count [1, 15]. These rare tumors are currently classified as anaplastic gangliogliomas and are assigned to WHO grade III. They show an unfavorable outcome [1, 15].

Here, we report of a malignant tumor of the cervical spinal cord in a 28-year old male patient with morphology of anaplastic ganglioglioma but genotype of diffuse midline glioma H3 K27M mutant.

## Clinical summary

**Fig. 1:**
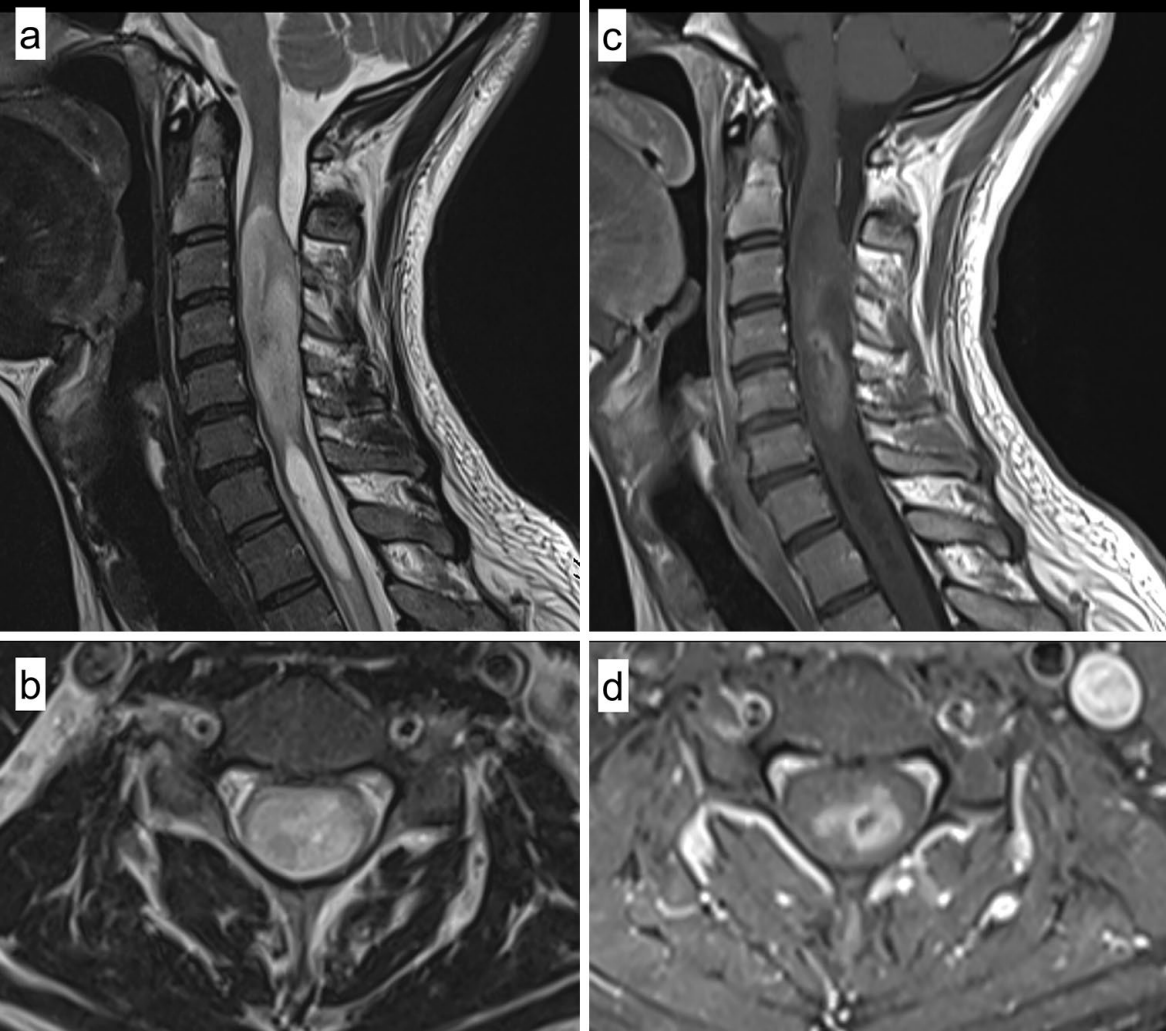
Radiological findings. Sagittal (**a**) and axial (**b**) T2 weighted magnetic resonance imaging (MRI) showing an inhomogeneous hyperintense intramedullary tumor in the upper cervical cord with a consecutive syringomyelia below the lesion. Sagittal (**c**) and axial (**d**) postcontrast T1 weighted imaging demonstrating a mild and inhomogeneous contrast enhancement in the central parts of the lesion with minimal central hemorrhage (without hemosiderin capping)

A 28-year old male Caucasian presented with neck and shoulder pain, dysesthesia of all four limbs and hypesthesia affecting both hands, without motor deficits. Magnetic resonance imaging (MRI) showed an intradural, intramedullary mass of the cervical spinal cord at levels C2–C6 of 6.4 cm length and 1.7 cm diameter with distinct expansion of the myelon. T2 weighted sequences showed an inhomogeneous, hyperintense lesion with a consecutive syringomyelia ranging from C6/7 to T1/2 with a length of 4 cm and a maximum diameter of 0.7 cm (Fig. 1a, b). In T1 weighted imaging the lesion was iso- to hypointense with a mild to moderate, inhomogeneous contrast enhancement in the central parts without evidence of hemorrhage (hemosiderin capping), therefore primarily suspicious for glioma or ependymoma (Fig. 1c, d). Preoperative median nerve somatosensory evoked potentials (SSEP) were normal, with a deceleration of tibial nerve SSEPs on both sides indicating an impairment of spino-thalamo-cortical fibers.

The patient underwent surgical resection of the tumor through laminoplasty C2–C6 using intraoperative neurophysiological monitoring with SSEPs. Intraoperative findings showed a solid, grey and partially bleeding tumor mass with infiltration of the surrounding myelon. Therefore, after intraoperative deterioration of evoked potentials a complete resection could not be achieved. Postoperative MRI scans showed a partial resection with minimal residual contrast enhancement in T1 images and residual T2-hyperintense tumor lesions. In postoperative clinical examination the patient showed an incomplete cross-sectional sensorimotor syndrome from C4 that gradually declined under physical therapy. Concomitant radiochemotherapy with Temozolomide analogous to STUPP protocol [16] was initiated after diagnosis with a local application of 28 × 1.8 Gy including residual tumor volume and high risk areas.

## Pathological findings

**Fig. 2:**
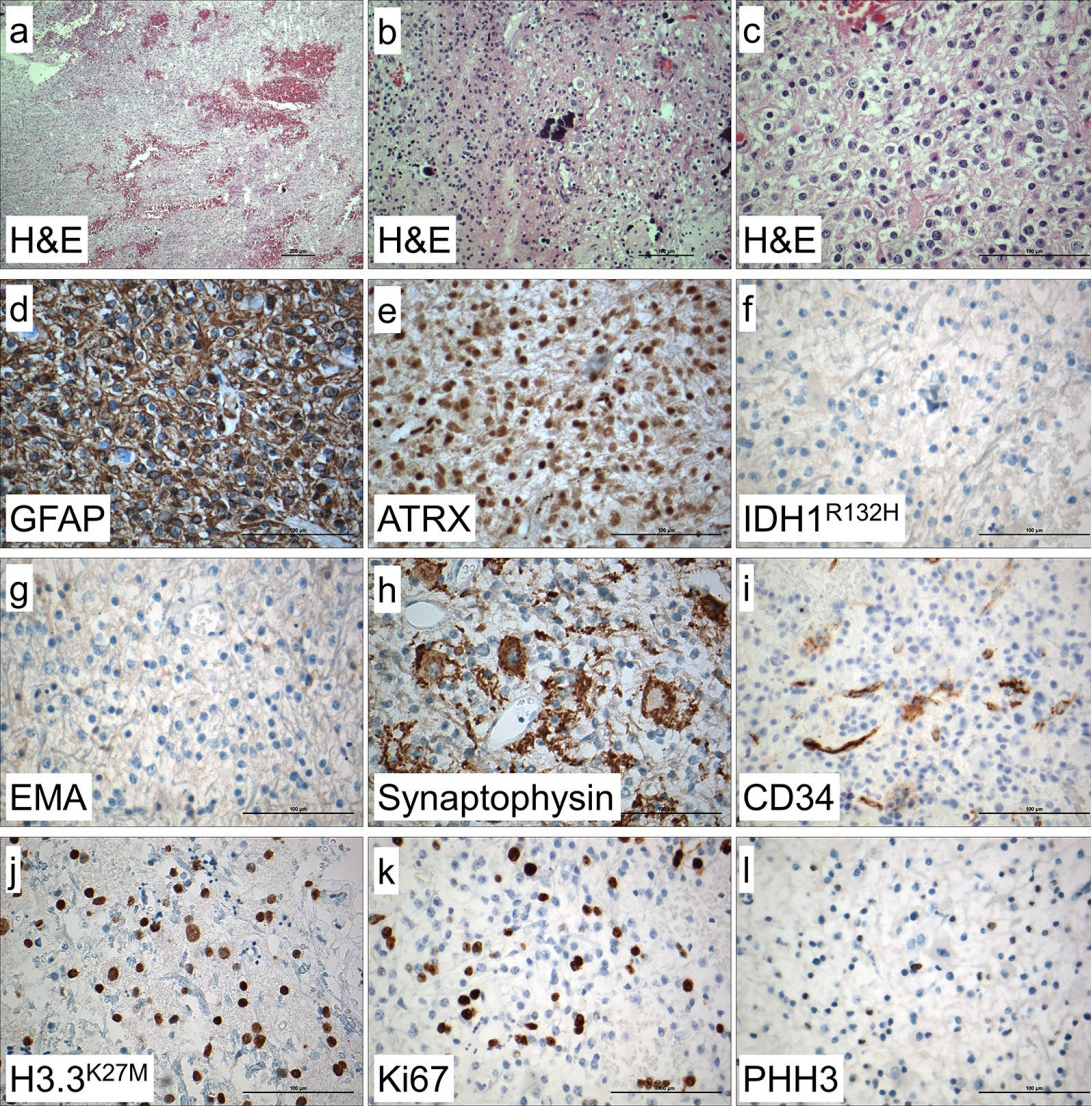
Histological and immunohistochemical findings. In H&E stained sections, mildly to highly pleomorphic glial tumor cells with glial cell processes and hemorrhages were detected (**a**). Calcifications and very round tumor cells with only short processes and perinuclear halos (**b**), intermingled within neuronal cells showing dysmorphic appearance, were also visible (**c**). Immunohistochemistry showed tumor cells being positive for GFAP (**d**) with retained ATRX expression (**e**), and no expression of IDH1 R132H mutant protein (**f**). Tumor cells were negative for EMA (**g**). Ganglionic cells were positive for synaptophysin (**h**) and there were some CD34 positive tumor cells (**i**). Histone H3.3 K27M mutant protein was strongly expressed in the nuclei of tumor cells (**j**). Ki67 index showed 20% positive cells (**k**) and there were some PHH3 (H3S10p) positive cells (**l**). Magnification: **a**: 10 ×, **b**: 20 ×, **c–l**: 40 ×

Histologically, the H&E staining showed a pleomorphic glial tumor with calcifications (Fig. 2a-c). Partially, the tumor cells were very round with only short processes and perinuclear halos (Fig. 2b), and mitotic activity was increased. Frequently, there were neuronal cells with multiple nuclei intermingled within glial tumor cells (Fig. 2c).

Immunohistochemistry performed on a Ventana Benchmark Ultra System with standard protocols showed that glial tumor cells were positive for GFAP (glial fibrillary acidic protein, Fig. 2d). Nuclear expression of ATRX (nuclear immunopositivity for α-thalassemia/mental-retardation-syndrome-X-linked) was retained (Fig. 2e) and there was no expression of IDH1 (isocitrate dehydrogenase 1) R132H mutant protein (Fig. 2f). Tumor cells were negative for EMA (Fig. 2g). Neuronal cells were positive for synaptophysin (Fig. 2h) and some tumor cells showing positivity for CD34 (Fig. 2i). Immunohistochemistry with an antibody against mutant histone H3.3 K27M (Fig. 2j) showed nuclear staining in most of the tumor cells. Proliferation was increased with 20% Ki67 positive cells (Fig. 2k) and some PHH3 (phosphorylated histone H3, H3S10p) positive cells (Fig. 2l).

**Figure. 3:**
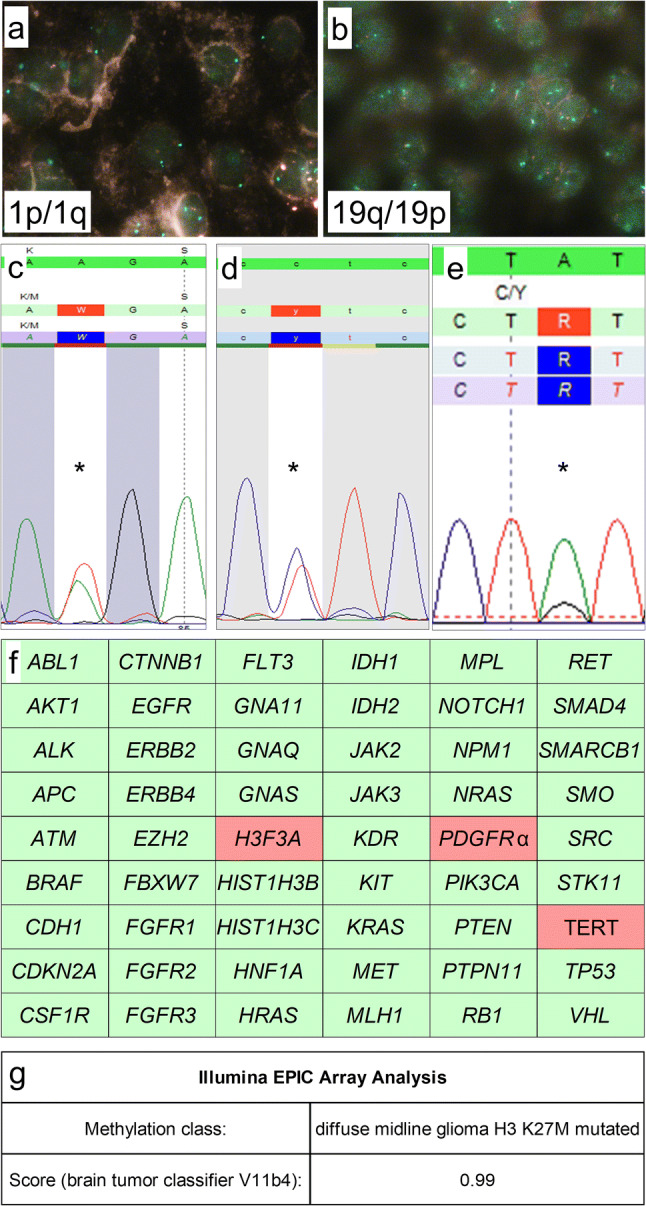
Molecular genetic findings. FISH-analysis showed retained 1p (**a**) and 19q (**b**) expression. Mutation analysis using Sanger sequencing showed a *H3F3A* K27M mutation (**c**) and a TERT C228T promoter mutation (**d**), NGS showed a *PDGFRα* Y849C mutation (**e**). Further molecular analysis showed wild-type status of all other 51 genes including *BRAF*, *HIST1H3B*, *HIST1H3C*, *IDH1*, and *IDH2* status (**f**). Epigenome-wide methylation profiling using the Illumina EPIC Array at the Department of Neuropathology, University Hospital Heidelberg/German Cancer Research Center, allocated the tumor to the methylation class of diffuse midline glioma H3 K27M mutant (**g**). Magnification: **a**, **b**: 100 × oil immersion. **c–e**: mutations are indicated using “*”. **f**: wild-type gene status is indicated by green, mutant gene status by red color

Molecular genetic analysis of the 1p and 19q status was performed by fluorescence in situ hybridization (FISH) using standard protocols. Analysis showed 16 out of 100 cells with a loss of chromosomal information on 1p compared with 1q (Fig. 3a) and eight out of 100 cells with a loss of chromosomal information on 19q compared to 19p (Fig. 3b). Thus, there was no evidence for a combined 1p/19q loss within the tumor.

Analysis of gene mutations was performed by DNA extraction using the Maxwell system (Promega) according to the manufacturer’s protocol and subsequent application of the AmpliSeq for Illumina Cancer Hotspot Panel v2 (Illumina) on an Illumina MiniSeq next generation sequencing device (Illumina) according to the manufacturer’s protocols. Additionally, Sanger sequencing on hot spot loci of TERT promoter, *H3F3A*, *HIST1H3B* and *HIST1H3C* genes was performed.

Mutational analysis confirmed the *H3F3A* (K27M) mutation (Fig. 3c) and revealed a TERT (C228T) promoter (Fig. 3d) and *PDGFRα* (Y849C) gene mutation (Fig. 3e). There was no mutation at the hot-spot regions of all other 51 genes investigated including *BRAF*, *HISH1H3B*, *HIST1H3C*, *IDH1*, and *IDH2* (Fig. 3f).

Additionally, epigenome-wide methylation profiling was performed at the Department of Neuropathology, University Hospital Heidelberg/German Cancer Research Center (DKFZ), using Illumina EPIC methylation bead chips [9]. This procedure enables to assign brain tumors according to their methylome to distinct molecular entities [9]. Well in line with the detection of the *H3F3A* K27M mutation, the tumor was allocated to the methylation class of diffuse midline glioma H3 K27M mutant (brain tumor classifier V11b4, Fig. 3g).

## Discussion

Diffuse midline gliomas H3 K27M mutant are rare tumors that typically arise within midline structures and the brain stem in younger patients [1, 2, 8]. These tumors show a devastating prognosis [1]. Diffuse midline gliomas have been shown by performing epigenome-wide methylation profiling to constitute a distinct methylation class [2]. Interestingly, diffuse midline gliomas show a broad range of phenotypes mimicking glioblastomas, primitive neuroectodermal tumors, medulloblastomas or even low-grade gliomas and glio-neuronal tumors [2, 9]. In contrast to the highly variable histological phenotype diffuse midline gliomas show a distinct genotype carrying the K27M mutation within the histone genes *H3F3A*, *HIST1H3B* or *HIST1H3C* [2, 8, 9].

In the case presented here, a tumor of the cervical spinal cord with histological appearance of ganglioglioma was found. Since mitotic activity was significantly increased, the tumor fulfilled the histological criteria for anaplastic ganglioglioma WHO Grade III (Fig. 2). Well in line with histology, the tumor showed expression of both glial (e.g. GFAP) and neuronal markers (e.g. synaptophysin) with pathological expression of CD34 and retained ATRX expression (Fig. 2). Proliferative activity assessed by Ki67 immunohistochemistry was significantly increased up to 20% (Fig. 2). Molecular workup showed no *IDH1, IDH2, and BRAF* mutations but revealed *H3F3A* K27M, TERT promoter (C228T) and *PDGFRα* Y849C mutations (Fig. 3).

Thus, the tumor showed the molecular hallmark of diffuse midline glioma H3 K27M mutant [1]. Well in line with this diagnosis was the epigenome-wide methylation analysis, allotting the tumor to the methylation class of diffuse midline glioma. Interestingly, additional *PDGFRα* Y849C and TERT promoter (C228T) mutations have been detected. While neither of these mutations have been described frequently in anaplastic ganglioglioma or diffuse midline glioma H3 K27M mutant, they both may point towards higher malignancy: The Y849 position of *PDGFRα* is of functional importance [17] and aberrant expression of *PDGFRα* [18] and TERT promoter mutations typically are frequently observed in glioblastoma, however, TERT promoter mutations also occur very frequently in oligodendroglioma WHO grade II [19, 20].

To our knowledge, this is the first reported case of a diffuse midline glioma H3 K27M mutated phenotypically mimicking anaplastic ganglioglioma that was confirmed by methylome profiling and carrying further TERT, and *PDGFRα* mutations.

A literature search revealed ten cases of phenotypical gangliogliomas WHO Grade I and three cases of anaplastic gangliogliomas WHO Grade III carrying the H3 K27M genotype [21–25]. Interestingly, seven of these ten gangliogliomas showed carried a *BRAF* V600E mutation [22]. All previously reported phenotypical anaplastic gangliogliomas WHO Grade III with H3 K27M genotype lacked *BRAF* V600E mutation [22]. This may indicate that some diffuse midline gliomas H3 K27M mutant are misinterpreted as anaplastic ganglioglioma. However, no methylome analyses has been performed in those cases. In a few cases double mutations of *H3F3A* K27M and *BRAF* V600E have been described [26, 27]. No systematic epigenome-wide methylation profiling of such tumors has been performed to date.

Longer survival in tumors with both *BRAF* V600E and H3 K27M mutations however suggest that these tumors should be separated from canonical diffuse glioma H3 K27M mutant [27]. Our case emphasis the importance of an integrated molecular workup including methylome profiling in rare and not clearly defined tumor entities.

In summary, the case presented here is a very rare example of diffuse midline glioma H3 K27M mutant phenotypically mimicking anaplastic ganglioglioma. It seems that the phenotypes of ganglioglioma and anaplastic ganglioglioma in parts represent heterogeneous groups of tumors in terms of their molecular background. Thus, there is the urgent need for combining both histology and molecular pathology including methylome analysis for advanced integrated diagnosis in glial and glioneuronal tumors. In an analogous manner to the introduction of integrated diagnosis in glioma with the 2016 WHO classification of tumors of the CNS [1] this will enable a refined patient care by introducing new individualized treatment strategies including targeted antibody therapies.
